# 16-Isopropyl-5,9-dimethyl­tetra­cyclo­[10.2.2.0^1,10^.0^4,9^]hexa­dec-15-ene-5,13,14-trimethanol ethanol monosolvate

**DOI:** 10.1107/S1600536811012207

**Published:** 2011-04-07

**Authors:** Jian Li, Xiao-ping Rao, Shi-bin Shang, Yan-qing Gao

**Affiliations:** a Institute of Chemical Industry of Forest Products, Chinese Academy of Forestry, Nanjing, 210042, People’s Republic of China

## Abstract

The title compound, C_24_H_40_O_3_·C_2_H_6_O, is a substituted tetra­cyclo­[10.2.2.0^1,10^.0^4,9^]hexa­decane derivative obtained from the reduction of maleopimaric acid which was isolated from a maleic anhydride modified rosin. In the crystal, the triol mol­ecule and the ethanol solvent mol­ecule are linked by hydroxyl O—H⋯O hydrogen bonds, giving a two-dimensional network structure.

## Related literature

For the isolation of maleic anhydride modified rosin, see: Halbrook & Lawrence (1958 ▶). For the crystal structure of maleopimaric acid, see: Rao *et al.* (2008 ▶).
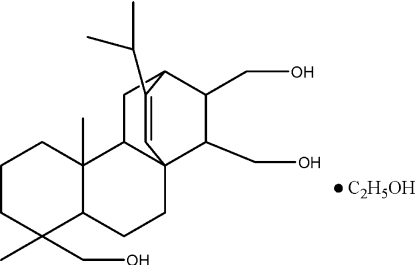


## Experimental

### 

#### Crystal data

C_24_H_40_O_3_·C_2_H_6_O
*M* *_r_* = 422.63
Orthorhombic, 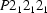

*a* = 9.1440 (18) Å
*b* = 9.6570 (19) Å
*c* = 28.073 (6) Å
*V* = 2478.9 (9) Å^3^
*Z* = 4
Mo *K*α radiation
μ = 0.07 mm^−1^
*T* = 293 K
0.30 × 0.20 × 0.10 mm

#### Data collection

Enraf–Nonius CAD-4 four-circle diffractometer
Absorption correction: ψ scan (North *et al.*, 1968 ▶) *T*_min_ = 0.978, *T*_max_ = 0.993
4919 measured reflections
2615 independent reflections
1786 reflections with *I* > 2σ(*I*)
*R*_int_ = 0.076
3 standard reflections every 200 reflections  intensity decay: 1%

#### Refinement

*R*[*F* ^2^ > 2σ(*F* ^2^)] = 0.063
*wR*(*F* ^2^) = 0.180
*S* = 1.00
2615 reflections
271 parameters
1 restraint
H-atom parameters constrained
Δρ_max_ = 0.29 e Å^−3^
Δρ_min_ = −0.22 e Å^−3^

### 

Data collection: *CAD-4 Software* (Enraf–Nonius, 1989 ▶); cell refinement: *CAD-4 Software*; data reduction: *XCAD4* (Harms & Wocadlo, 1995 ▶); program(s) used to solve structure: *SHELXS97* (Sheldrick, 2008 ▶); program(s) used to refine structure: *SHELXL97* (Sheldrick, 2008 ▶); molecular graphics: *SHELXTL* (Sheldrick, 2008 ▶); software used to prepare material for publication: *SHELXL97*.

## Supplementary Material

Crystal structure: contains datablocks I, global. DOI: 10.1107/S1600536811012207/zs2101sup1.cif

Structure factors: contains datablocks I. DOI: 10.1107/S1600536811012207/zs2101Isup2.hkl

Additional supplementary materials:  crystallographic information; 3D view; checkCIF report

## Figures and Tables

**Table 1:** Hydrogen-bond geometry (Å, °)

*D*—H⋯*A*	*D*—H	H⋯*A*	*D*⋯*A*	*D*—H⋯*A*
O2—H2*A*⋯O3^i^	0.85	2.27	2.749 (5)	116
O3—H3*A*⋯O1^i^	0.82	1.97	2.758 (5)	160
O4—H4*C*⋯O2^ii^	0.85	1.90	2.704 (6)	157

Symmetry codes: (i) 
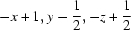
; (ii) 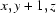.

**Fig. 1.**
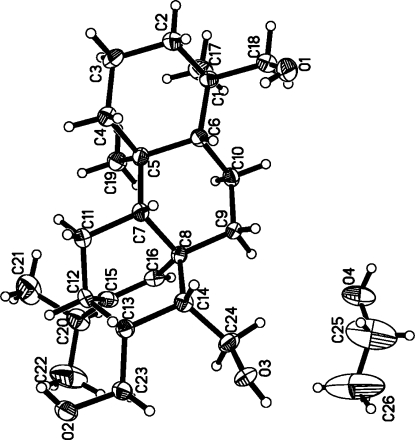
Molecular conformation and atom numbering scheme for (I), showing displacement ellipsoids at the 30% probability level.

**Fig. 2.**
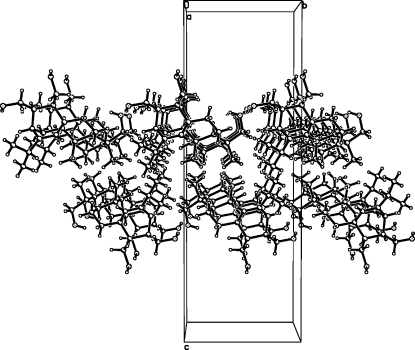
A view of the packing of the title compound in the unit cell, with hydrogen bonds shown as dashed lines.

## supplementary crystallographic information

### Comment

As an important natural resource, large amounts of rosin are available in China. Nowadays, rosin and its Diels-Alder adducts have been developed as a feedstock for synthesizing various chemicals and intermediates. Maleopimaric acid was isolated from maleic anhydride modified rosin (Halbrook & Lawrence, 1958), and its crystal structure has been reported (Rao *et al.*, 2008). The title compound C_24_H_40_O_3_ . C_2_H_6_O (I) was obtained on reduction of this tricarboxylic acid. The asymmetric unit comprises a triol molecule and an ethanol molecule of solvation (Fig. 1). In the crystal structure the molecules are linked by hydroxyl O–H···O hydrogen bonds and other interactions (Table 1, Fig. 2), giving a two-dimensional network structure. The absolute configurations for the 8 chiral centres in this molecule could not be assigned from the analysis: the configurations for the chosen enantiomer were C1(*R*), C5(*R*), C6(*R*), C7(*R*), C8(*S*), C12(*R*), C13(*R*), C14(*R*).

### Experimental

Maleopimaric acid (12.0 g) was dissolved in tetrahydrofuran (300 ml) at 263 K while stirring vigorously and lithium aluminium hydride (6.9 g) was added over a period of 2 h. The reaction was maintained for 1 h at reflux temperature after which water (6.9 ml) and 10% sodium hydroxide solution (6.9 ml) were added dropwise at 263 K. The mixture was filtered and the filtrate was concentrated, giving the crude product which was recrystallized with ethanol, giving the title compound. Crystals suitable for X-ray diffraction were obtained by slow evaporation of an ethanol solution.

### Refinement

All H atoms bonded to the C atoms were placed geometrically with C—H distances of 0.93–0.98 Å and included in the refinement in a riding motion approximation, with *U*_iso_(H) = 1.2 or 1.5*U*_eq_ of the carrier atom. All hydroxyl H atoms were placed geometrically at distances of 0.82–0.85 Å. The absolute configurations for the 8 chiral centres in this molecule could not be determined and the configurations for the chosen enantiomer were C1(*R*), C5(*R*), C6(*R*), C7(*R*), C8(*S*), C12(*R*), C13(*R*), C14(*R*).

### Crystal data

**Table:**

C_24_H_40_O_3_·C_2_H_6_O	*F*(000) = 936
*M**_r_* = 422.63	*D*_x_ = 1.132 Mg m^−^^3^
Orthorhombic, *P*2_1_2_1_2_1_	Mo *K*α radiation, λ = 0.71073 Å
Hall symbol: P 2ac 2ab	Cell parameters from 25 reflections
*a* = 9.1440 (18) Å	θ = 9–13°
*b* = 9.6570 (19) Å	µ = 0.07 mm^−^^1^
*c* = 28.073 (6) Å	*T* = 293 K
*V* = 2478.9 (9) Å^3^	Rod, colourless
*Z* = 4	0.30 × 0.20 × 0.10 mm

### Data collection

**Table:**

Enraf–Nonius CAD-4 four-circle diffractometer	1786 reflections with *I* > 2σ(*I*)
Radiation source: fine-focus sealed tube	*R*_int_ = 0.076
graphite	θ_max_ = 25.4°, θ_min_ = 1.5°
ω–2θ scans	*h* = −11→0
Absorption correction: ψ scan (North *et al.*, 1968)	*k* = −11→11
*T*_min_ = 0.978, *T*_max_ = 0.993	*l* = −33→0
4919 measured reflections	3 standard reflections every 200 reflections
2615 independent reflections	intensity decay: 1%

### Refinement

**Table:**

Refinement on *F*^2^	Primary atom site location: structure-invariant direct methods
Least-squares matrix: full	Secondary atom site location: difference Fourier map
*R*[*F*^2^ > 2σ(*F*^2^)] = 0.063	Hydrogen site location: inferred from neighbouring sites
*wR*(*F*^2^) = 0.180	H-atom parameters constrained
*S* = 1.00	*w* = 1/[σ^2^(*F*_o_^2^) + (0.10*P*)^2^ + 0.50*P*] where *P* = (*F*_o_^2^ + 2*F*_c_^2^)/3
2615 reflections	(Δ/σ)_max_ < 0.001
271 parameters	Δρ_max_ = 0.29 e Å^−^^3^
1 restraint	Δρ_min_ = −0.22 e Å^−^^3^

### Special details

**Table:**

Geometry. All e.s.d.'s (except the e.s.d. in the dihedral angle between two l.s. planes) are estimated using the full covariance matrix. The cell e.s.d.'s are taken into account individually in the estimation of e.s.d.'s in distances, angles and torsion angles; correlations between e.s.d.'s in cell parameters are only used when they are defined by crystal symmetry. An approximate (isotropic) treatment of cell e.s.d.'s is used for estimating e.s.d.'s involving l.s. planes.
Refinement. Refinement of *F*^2^ against ALL reflections. The weighted *R*-factor *wR* and goodness of fit *S* are based on *F*^2^, conventional *R*-factors *R* are based on *F*, with *F* set to zero for negative *F*^2^. The threshold expression of *F*^2^ > σ(*F*^2^) is used only for calculating *R*-factors(gt) *etc*. and is not relevant to the choice of reflections for refinement. *R*-factors based on *F*^2^ are statistically about twice as large as those based on *F*, and *R*- factors based on ALL data will be even larger.

### Fractional atomic coordinates and isotropic or equivalent isotropic displacement parameters (Å^2^)

**Table:**

	*x*	*y*	*z*	*U*_iso_*/*U*_eq_	
O1	0.1259 (4)	0.9737 (4)	0.16607 (12)	0.0551 (10)	
H1A	0.1038	0.8921	0.1748	0.083*	
C1	0.1816 (5)	0.8655 (5)	0.08762 (16)	0.0379 (12)	
O2	0.6060 (4)	0.0733 (3)	0.19112 (12)	0.0476 (9)	
H2A	0.5244	0.0514	0.1787	0.071*	
C2	0.0246 (6)	0.8104 (5)	0.08231 (19)	0.0480 (14)	
H2B	−0.0204	0.8058	0.1136	0.058*	
H2C	−0.0315	0.8752	0.0632	0.058*	
O3	0.6437 (4)	0.4603 (4)	0.27068 (12)	0.0512 (9)	
H3A	0.7228	0.4721	0.2839	0.077*	
C3	0.0171 (6)	0.6667 (6)	0.05903 (19)	0.0491 (14)	
H3B	−0.0838	0.6358	0.0578	0.059*	
H3C	0.0538	0.6719	0.0267	0.059*	
C4	0.1076 (5)	0.5638 (5)	0.08736 (18)	0.0435 (12)	
H4A	0.1013	0.4738	0.0721	0.052*	
H4B	0.0661	0.5551	0.1190	0.052*	
C5	0.2677 (5)	0.6040 (5)	0.09188 (16)	0.0335 (11)	
C6	0.2777 (5)	0.7538 (4)	0.11185 (16)	0.0329 (11)	
H6A	0.2382	0.7466	0.1442	0.039*	
C7	0.3387 (5)	0.5085 (4)	0.12991 (16)	0.0330 (10)	
H7A	0.2768	0.5169	0.1583	0.040*	
C8	0.4957 (5)	0.5467 (4)	0.14670 (14)	0.0290 (10)	
C9	0.5092 (5)	0.7028 (5)	0.15633 (17)	0.0361 (11)	
H9A	0.4657	0.7229	0.1871	0.043*	
H9B	0.6121	0.7266	0.1582	0.043*	
C10	0.4373 (5)	0.7937 (5)	0.11907 (17)	0.0365 (11)	
H10A	0.4894	0.7846	0.0891	0.044*	
H10B	0.4430	0.8898	0.1290	0.044*	
C11	0.3361 (5)	0.3518 (4)	0.11655 (18)	0.0390 (12)	
H11A	0.2590	0.3054	0.1341	0.047*	
H11B	0.3160	0.3415	0.0828	0.047*	
C12	0.4843 (6)	0.2851 (5)	0.12847 (18)	0.0396 (12)	
H12A	0.4837	0.1867	0.1200	0.048*	
C13	0.5099 (5)	0.3032 (5)	0.18254 (16)	0.0345 (11)	
H13A	0.4229	0.2675	0.1988	0.041*	
C14	0.5201 (5)	0.4612 (5)	0.19389 (15)	0.0343 (11)	
H14A	0.4356	0.4818	0.2142	0.041*	
C15	0.6011 (6)	0.3603 (5)	0.10101 (17)	0.0418 (12)	
C16	0.6064 (5)	0.4944 (5)	0.11130 (16)	0.0369 (11)	
H16A	0.6748	0.5530	0.0974	0.044*	
C17	0.2395 (7)	0.9152 (6)	0.03954 (18)	0.0601 (16)	
H17A	0.2460	0.8381	0.0180	0.090*	
H17B	0.3346	0.9554	0.0437	0.090*	
H17C	0.1741	0.9834	0.0266	0.090*	
C18	0.1740 (6)	0.9966 (5)	0.11865 (19)	0.0495 (13)	
H18A	0.2704	1.0385	0.1197	0.059*	
H18B	0.1084	1.0622	0.1036	0.059*	
C19	0.3445 (6)	0.5886 (5)	0.04277 (16)	0.0466 (13)	
H19A	0.3362	0.4945	0.0321	0.070*	
H19B	0.4459	0.6126	0.0458	0.070*	
H19C	0.2988	0.6490	0.0201	0.070*	
C20	0.6958 (7)	0.2905 (6)	0.06440 (19)	0.0571 (15)	
H20A	0.7557	0.3625	0.0494	0.069*	
C21	0.5967 (10)	0.2283 (7)	0.0251 (2)	0.086 (2)	
H21A	0.5324	0.2987	0.0131	0.129*	
H21B	0.5399	0.1539	0.0382	0.129*	
H21C	0.6564	0.1937	−0.0004	0.129*	
C22	0.7994 (9)	0.1842 (9)	0.0851 (2)	0.108 (3)	
H22A	0.8570	0.2262	0.1098	0.161*	
H22B	0.8628	0.1502	0.0605	0.161*	
H22C	0.7443	0.1087	0.0982	0.161*	
C23	0.6387 (6)	0.2176 (5)	0.19917 (18)	0.0400 (11)	
H23A	0.7259	0.2438	0.1817	0.048*	
H23B	0.6563	0.2338	0.2328	0.048*	
C24	0.6531 (6)	0.5052 (5)	0.22226 (17)	0.0460 (13)	
H24A	0.7403	0.4664	0.2078	0.055*	
H24B	0.6616	0.6053	0.2214	0.055*	
O4	0.8584 (5)	0.9317 (6)	0.20316 (18)	0.0869 (15)	
H4C	0.7882	0.9888	0.2061	0.130*	
C25	0.8793 (10)	0.8696 (12)	0.2457 (4)	0.157 (5)	
H25A	0.7847	0.8333	0.2551	0.189*	
H25B	0.9021	0.9432	0.2680	0.189*	
C26	0.9818 (13)	0.7624 (13)	0.2547 (6)	0.222 (8)	
H26A	0.9793	0.7386	0.2879	0.333*	
H26B	1.0782	0.7937	0.2464	0.333*	
H26C	0.9574	0.6826	0.2360	0.333*	

### Atomic displacement parameters (Å^2^)

**Table:**

	*U*^11^	*U*^22^	*U*^33^	*U*^12^	*U*^13^	*U*^23^
O1	0.055 (2)	0.058 (2)	0.052 (2)	0.006 (2)	−0.0004 (18)	−0.0159 (18)
C1	0.045 (3)	0.034 (2)	0.034 (3)	0.006 (2)	−0.008 (2)	0.004 (2)
O2	0.045 (2)	0.0382 (18)	0.059 (2)	0.0058 (18)	−0.0007 (18)	0.0046 (16)
C2	0.051 (3)	0.044 (3)	0.049 (3)	0.011 (3)	−0.013 (3)	−0.003 (2)
O3	0.055 (2)	0.057 (2)	0.042 (2)	0.005 (2)	−0.0229 (17)	0.0004 (17)
C3	0.035 (3)	0.058 (3)	0.054 (3)	−0.001 (3)	−0.014 (2)	−0.006 (3)
C4	0.043 (3)	0.039 (3)	0.049 (3)	−0.002 (2)	−0.008 (2)	0.000 (2)
C5	0.036 (3)	0.033 (2)	0.032 (2)	−0.002 (2)	−0.003 (2)	−0.003 (2)
C6	0.039 (3)	0.031 (2)	0.028 (2)	0.002 (2)	0.000 (2)	0.001 (2)
C7	0.036 (2)	0.028 (2)	0.036 (2)	−0.002 (2)	−0.002 (2)	−0.0007 (19)
C8	0.031 (2)	0.030 (2)	0.025 (2)	−0.001 (2)	0.0003 (19)	−0.0025 (19)
C9	0.036 (3)	0.031 (2)	0.041 (3)	0.000 (2)	−0.011 (2)	−0.005 (2)
C10	0.042 (3)	0.028 (2)	0.040 (3)	−0.003 (2)	0.000 (2)	−0.001 (2)
C11	0.041 (3)	0.031 (2)	0.046 (3)	−0.001 (2)	−0.005 (2)	−0.004 (2)
C12	0.048 (3)	0.024 (2)	0.047 (3)	0.003 (2)	−0.007 (2)	−0.008 (2)
C13	0.034 (3)	0.032 (2)	0.038 (3)	−0.002 (2)	−0.001 (2)	0.003 (2)
C14	0.036 (3)	0.036 (2)	0.030 (2)	0.002 (2)	0.004 (2)	−0.001 (2)
C15	0.045 (3)	0.043 (3)	0.037 (3)	0.014 (2)	−0.004 (2)	0.002 (2)
C16	0.031 (2)	0.043 (3)	0.037 (3)	0.003 (2)	0.000 (2)	0.006 (2)
C17	0.078 (4)	0.055 (3)	0.047 (3)	0.013 (3)	−0.001 (3)	0.017 (3)
C18	0.049 (3)	0.041 (3)	0.059 (3)	0.008 (2)	−0.009 (3)	−0.002 (3)
C19	0.056 (3)	0.049 (3)	0.035 (3)	0.009 (3)	−0.005 (2)	−0.004 (2)
C20	0.071 (4)	0.056 (3)	0.044 (3)	0.014 (3)	0.011 (3)	−0.001 (3)
C21	0.125 (7)	0.080 (5)	0.053 (4)	0.012 (5)	0.017 (4)	−0.022 (4)
C22	0.125 (7)	0.125 (7)	0.073 (5)	0.082 (6)	0.028 (5)	0.005 (5)
C23	0.044 (3)	0.036 (2)	0.040 (3)	0.005 (2)	−0.003 (2)	0.002 (2)
C24	0.049 (3)	0.049 (3)	0.040 (3)	−0.003 (3)	−0.016 (2)	0.004 (2)
O4	0.058 (3)	0.100 (3)	0.103 (4)	0.033 (3)	0.011 (3)	0.024 (3)
C25	0.092 (7)	0.185 (11)	0.194 (11)	0.032 (8)	0.064 (8)	0.100 (10)
C26	0.098 (8)	0.212 (15)	0.356 (18)	−0.024 (9)	−0.054 (10)	0.189 (14)

### Geometric parameters (Å, °)

**Table:**

O1—C18	1.419 (6)	C12—C13	1.546 (6)
O1—H1A	0.8500	C12—H12A	0.9800
C1—C17	1.527 (7)	C13—C23	1.512 (6)
C1—C18	1.538 (7)	C13—C14	1.561 (6)
C1—C2	1.539 (7)	C13—H13A	0.9800
C1—C6	1.549 (6)	C14—C24	1.515 (6)
O2—C23	1.444 (6)	C14—H14A	0.9800
O2—H2A	0.8500	C15—C16	1.327 (7)
C2—C3	1.536 (7)	C15—C20	1.504 (7)
C2—H2B	0.9700	C16—H16A	0.9300
C2—H2C	0.9700	C17—H17A	0.9600
O3—C24	1.429 (6)	C17—H17B	0.9600
O3—H3A	0.8200	C17—H17C	0.9600
C3—C4	1.518 (7)	C18—H18A	0.9700
C3—H3B	0.9700	C18—H18B	0.9700
C3—H3C	0.9700	C19—H19A	0.9600
C4—C5	1.521 (7)	C19—H19B	0.9600
C4—H4A	0.9700	C19—H19C	0.9600
C4—H4B	0.9700	C20—C22	1.513 (9)
C5—C7	1.553 (6)	C20—C21	1.549 (9)
C5—C6	1.553 (6)	C20—H20A	0.9800
C5—C19	1.554 (6)	C21—H21A	0.9600
C6—C10	1.523 (6)	C21—H21B	0.9600
C6—H6A	0.9800	C21—H21C	0.9600
C7—C8	1.555 (6)	C22—H22A	0.9600
C7—C11	1.559 (6)	C22—H22B	0.9600
C7—H7A	0.9800	C22—H22C	0.9600
C8—C16	1.505 (6)	C23—H23A	0.9700
C8—C9	1.537 (6)	C23—H23B	0.9700
C8—C14	1.577 (6)	C24—H24A	0.9700
C9—C10	1.516 (6)	C24—H24B	0.9700
C9—H9A	0.9700	O4—C25	1.349 (10)
C9—H9B	0.9700	O4—H4C	0.8500
C10—H10A	0.9700	C25—C26	1.420 (9)
C10—H10B	0.9700	C25—H25A	0.9700
C11—C12	1.538 (7)	C25—H25B	0.9700
C11—H11A	0.9700	C26—H26A	0.9600
C11—H11B	0.9700	C26—H26B	0.9600
C12—C15	1.504 (7)	C26—H26C	0.9600
			
C18—O1—H1A	119.2	C12—C13—C14	108.6 (4)
C17—C1—C18	104.9 (4)	C23—C13—H13A	107.2
C17—C1—C2	110.3 (4)	C12—C13—H13A	107.2
C18—C1—C2	107.3 (4)	C14—C13—H13A	107.2
C17—C1—C6	114.3 (4)	C24—C14—C13	115.4 (4)
C18—C1—C6	110.5 (4)	C24—C14—C8	114.1 (4)
C2—C1—C6	109.3 (4)	C13—C14—C8	109.4 (3)
C23—O2—H2A	119.1	C24—C14—H14A	105.7
C3—C2—C1	113.3 (4)	C13—C14—H14A	105.7
C3—C2—H2B	108.9	C8—C14—H14A	105.7
C1—C2—H2B	108.9	C16—C15—C20	124.5 (5)
C3—C2—H2C	108.9	C16—C15—C12	112.6 (5)
C1—C2—H2C	108.9	C20—C15—C12	122.8 (4)
H2B—C2—H2C	107.7	C15—C16—C8	116.5 (5)
C24—O3—H3A	109.5	C15—C16—H16A	121.7
C4—C3—C2	110.2 (4)	C8—C16—H16A	121.7
C4—C3—H3B	109.6	C1—C17—H17A	109.5
C2—C3—H3B	109.6	C1—C17—H17B	109.5
C4—C3—H3C	109.6	H17A—C17—H17B	109.5
C2—C3—H3C	109.6	C1—C17—H17C	109.5
H3B—C3—H3C	108.1	H17A—C17—H17C	109.5
C3—C4—C5	113.7 (4)	H17B—C17—H17C	109.5
C3—C4—H4A	108.8	O1—C18—C1	114.7 (4)
C5—C4—H4A	108.8	O1—C18—H18A	108.6
C3—C4—H4B	108.8	C1—C18—H18A	108.6
C5—C4—H4B	108.8	O1—C18—H18B	108.6
H4A—C4—H4B	107.7	C1—C18—H18B	108.6
C4—C5—C7	107.9 (4)	H18A—C18—H18B	107.6
C4—C5—C6	108.9 (4)	C5—C19—H19A	109.5
C7—C5—C6	106.3 (3)	C5—C19—H19B	109.5
C4—C5—C19	109.7 (4)	H19A—C19—H19B	109.5
C7—C5—C19	111.3 (4)	C5—C19—H19C	109.5
C6—C5—C19	112.5 (4)	H19A—C19—H19C	109.5
C10—C6—C1	115.2 (4)	H19B—C19—H19C	109.5
C10—C6—C5	109.9 (4)	C15—C20—C22	113.7 (5)
C1—C6—C5	117.2 (4)	C15—C20—C21	108.9 (5)
C10—C6—H6A	104.3	C22—C20—C21	112.1 (6)
C1—C6—H6A	104.3	C15—C20—H20A	107.2
C5—C6—H6A	104.3	C22—C20—H20A	107.2
C5—C7—C8	116.9 (4)	C21—C20—H20A	107.2
C5—C7—C11	113.9 (4)	C20—C21—H21A	109.5
C8—C7—C11	108.5 (4)	C20—C21—H21B	109.5
C5—C7—H7A	105.5	H21A—C21—H21B	109.5
C8—C7—H7A	105.5	C20—C21—H21C	109.5
C11—C7—H7A	105.5	H21A—C21—H21C	109.5
C16—C8—C9	113.0 (4)	H21B—C21—H21C	109.5
C16—C8—C7	109.9 (3)	C20—C22—H22A	109.5
C9—C8—C7	111.1 (4)	C20—C22—H22B	109.5
C16—C8—C14	106.5 (3)	H22A—C22—H22B	109.5
C9—C8—C14	110.8 (4)	C20—C22—H22C	109.5
C7—C8—C14	105.1 (3)	H22A—C22—H22C	109.5
C10—C9—C8	114.3 (4)	H22B—C22—H22C	109.5
C10—C9—H9A	108.7	O2—C23—C13	108.5 (4)
C8—C9—H9A	108.7	O2—C23—H23A	110.0
C10—C9—H9B	108.7	C13—C23—H23A	110.0
C8—C9—H9B	108.7	O2—C23—H23B	110.0
H9A—C9—H9B	107.6	C13—C23—H23B	110.0
C9—C10—C6	111.1 (4)	H23A—C23—H23B	108.4
C9—C10—H10A	109.4	O3—C24—C14	111.5 (4)
C6—C10—H10A	109.4	O3—C24—H24A	109.3
C9—C10—H10B	109.4	C14—C24—H24A	109.3
C6—C10—H10B	109.4	O3—C24—H24B	109.3
H10A—C10—H10B	108.0	C14—C24—H24B	109.3
C12—C11—C7	109.9 (4)	H24A—C24—H24B	108.0
C12—C11—H11A	109.7	C25—O4—H4C	108.0
C7—C11—H11A	109.7	O4—C25—C26	125.2 (11)
C12—C11—H11B	109.7	O4—C25—H25A	106.0
C7—C11—H11B	109.7	C26—C25—H25A	106.0
H11A—C11—H11B	108.2	O4—C25—H25B	106.0
C15—C12—C11	108.2 (4)	C26—C25—H25B	106.0
C15—C12—C13	109.9 (4)	H25A—C25—H25B	106.3
C11—C12—C13	107.5 (4)	C25—C26—H26A	109.5
C15—C12—H12A	110.4	C25—C26—H26B	109.5
C11—C12—H12A	110.4	H26A—C26—H26B	109.5
C13—C12—H12A	110.4	C25—C26—H26C	109.5
C23—C13—C12	111.1 (4)	H26A—C26—H26C	109.5
C23—C13—C14	115.1 (4)	H26B—C26—H26C	109.5
			
C17—C1—C2—C3	75.7 (5)	C5—C7—C11—C12	137.0 (4)
C18—C1—C2—C3	−170.6 (4)	C8—C7—C11—C12	4.9 (5)
C6—C1—C2—C3	−50.7 (6)	C7—C11—C12—C15	−58.8 (5)
C1—C2—C3—C4	57.0 (6)	C7—C11—C12—C13	59.9 (5)
C2—C3—C4—C5	−58.6 (6)	C15—C12—C13—C23	−73.2 (5)
C3—C4—C5—C7	168.2 (4)	C11—C12—C13—C23	169.3 (4)
C3—C4—C5—C6	53.2 (5)	C15—C12—C13—C14	54.4 (5)
C3—C4—C5—C19	−70.3 (5)	C11—C12—C13—C14	−63.2 (5)
C17—C1—C6—C10	55.4 (5)	C23—C13—C14—C24	−3.2 (6)
C18—C1—C6—C10	−62.6 (5)	C12—C13—C14—C24	−128.5 (4)
C2—C1—C6—C10	179.5 (4)	C23—C13—C14—C8	127.1 (4)
C17—C1—C6—C5	−76.2 (6)	C12—C13—C14—C8	1.8 (5)
C18—C1—C6—C5	165.8 (4)	C16—C8—C14—C24	75.8 (5)
C2—C1—C6—C5	47.9 (5)	C9—C8—C14—C24	−47.4 (5)
C4—C5—C6—C10	177.1 (4)	C7—C8—C14—C24	−167.5 (4)
C7—C5—C6—C10	61.1 (5)	C16—C8—C14—C13	−55.2 (5)
C19—C5—C6—C10	−61.0 (5)	C9—C8—C14—C13	−178.4 (4)
C4—C5—C6—C1	−48.9 (5)	C7—C8—C14—C13	61.5 (4)
C7—C5—C6—C1	−165.0 (4)	C11—C12—C15—C16	59.2 (6)
C19—C5—C6—C1	72.9 (5)	C13—C12—C15—C16	−57.9 (5)
C4—C5—C7—C8	−170.1 (4)	C11—C12—C15—C20	−118.2 (5)
C6—C5—C7—C8	−53.3 (5)	C13—C12—C15—C20	124.7 (5)
C19—C5—C7—C8	69.6 (5)	C20—C15—C16—C8	176.0 (4)
C4—C5—C7—C11	62.0 (5)	C12—C15—C16—C8	−1.3 (6)
C6—C5—C7—C11	178.8 (4)	C9—C8—C16—C15	−179.8 (4)
C19—C5—C7—C11	−58.3 (5)	C7—C8—C16—C15	−55.0 (5)
C5—C7—C8—C16	−81.2 (5)	C14—C8—C16—C15	58.4 (5)
C11—C7—C8—C16	49.2 (5)	C17—C1—C18—O1	−178.4 (4)
C5—C7—C8—C9	44.7 (5)	C2—C1—C18—O1	64.3 (5)
C11—C7—C8—C9	175.1 (4)	C6—C1—C18—O1	−54.8 (6)
C5—C7—C8—C14	164.5 (4)	C16—C15—C20—C22	115.7 (7)
C11—C7—C8—C14	−65.0 (4)	C12—C15—C20—C22	−67.2 (8)
C16—C8—C9—C10	81.0 (5)	C16—C15—C20—C21	−118.4 (6)
C7—C8—C9—C10	−43.1 (6)	C12—C15—C20—C21	58.7 (6)
C14—C8—C9—C10	−159.6 (4)	C12—C13—C23—O2	−62.5 (5)
C8—C9—C10—C6	54.2 (6)	C14—C13—C23—O2	173.5 (4)
C1—C6—C10—C9	161.3 (4)	C13—C14—C24—O3	−71.8 (5)
C5—C6—C10—C9	−63.8 (5)	C8—C14—C24—O3	160.3 (4)

### Hydrogen-bond geometry (Å, °)

**Table:**

*D*—H···*A*	*D*—H	H···*A*	*D*···*A*	*D*—H···*A*
O1—H1A···O4^i^	0.85	2.41	2.689 (6)	100
O2—H2A···O3^ii^	0.85	2.27	2.749 (5)	116
O3—H3A···O1^ii^	0.82	1.97	2.758 (5)	160
O4—H4C···O2^iii^	0.85	1.90	2.704 (6)	157
C2—H2B···O1	0.97	2.57	2.979 (6)	106
C6—H6A···O1	0.98	2.50	2.959 (6)	108
C12—H12A···O2	0.98	2.54	2.918 (6)	103
C23—H23B···O3	0.97	2.43	3.086 (6)	124

Symmetry codes: (i) *x*−1, *y*, *z*; (ii) −*x*+1, *y*−1/2, −*z*+1/2; (iii) *x*, *y*+1, *z*.

## References

[bb1] Enraf–Nonius (1989). *CAD-4 Software* Enraf–Nonius, Delft, The Netherlands.

[bb2] Halbrook, N. J. & Lawrence, R. V. (1958). *J. Am. Chem. Soc.* **80**, 368–370.

[bb3] Harms, K. & Wocadlo, S. (1995). *XCAD4* University of Marburg, Germany.

[bb4] North, A. C. T., Phillips, D. C. & Mathews, F. S. (1968). *Acta Cryst.* A**24**, 351–359.

[bb5] Rao, X. P., Song, Z. Q., Yao, Y. J., Han, C. R. & Shang, S. B. (2008). *Nat. Prod. Res.* **22**, 854–859. 10.1080/14786410701640585 18626819

[bb6] Sheldrick, G. M. (2008). *Acta Cryst.* A**64**, 112–122. 10.1107/S0108767307043930 18156677

